# Thromboembolic Diseases Among Intensive Care Unit Patients in Al-Qassim Region, Saudi Arabia

**DOI:** 10.7759/cureus.33033

**Published:** 2022-12-28

**Authors:** Ayman Harbi, Abdullah N AlSamani, Nouf M Adawi, Rabab Alswyan, Maha M AlDhilan, Saleh A Alajlan, Khalid A Alomary

**Affiliations:** 1 Department of Medicine, Qassim University, Buraydah, SAU; 2 Department of Radiology, Qassim University, Buraydah, SAU; 3 Department of Gynecology, Qassim University, Buraydah, SAU; 4 Department of Surgery, Qassim University, Buraydah, SAU; 5 Department of Medicine, Ministry of Health, Buraydah, SAU; 6 Department of Internal Medicine, King Fahad Specialist Hospital (KFSH), Buraydah, SAU; 7 Department of Critical Care Medicine, Ministry of Health, Buraydah, SAU

**Keywords:** deep vein thrombosis (dvt), pulmonary embolism, deep vein thrombosis, icu patients, thrombotic events

## Abstract

Background

Venous and arterial thrombotic conditions are the two types of thromboembolic events. Main venous thromboembolism (VTE) includes deep vein thrombosis (DVT) and pulmonary embolism (PE), while arterial thromboses include ischemic stroke and ischemic heart disease (IHD).

Aim

This study aimed to assess the prevalence of thromboembolic events among intensive care unit (ICU) patients in Al-Qassim region, Saudi Arabia.

Patients and methods

This is a retrospective chart review of ICU patients diagnosed with thromboembolic disease who were seen at the intensive care unit of King Fahad Specialist Hospital between July 2020 and June 2022. Data were obtained from hospital medical files and gathered into an Excel sheet (Microsoft Corp., Redmond, WA, USA). All data analyses were carried out using Statistical Package for the Social Sciences (SPSS) version 26 (IBM SPSS Statistics, Armonk, NY, USA).

Results

Of the 38 patients included, 52.6% were males (mean age: 60.7; standard deviation (SD): 23.9). The most common risk factors for thromboembolic events were immobilization (23.7%) and major surgeries (18.4%). The incidence of DVT was 42.1%, while PE was 39.5%. Seven patients were detected with combined incidence (DVT and PE). Mortality rates accounted for 39.5%. It is interesting to note that the prevalence of patients who use heparin treatment was statistically significantly higher among DVT patients (p=0.043).

Conclusion

The incidence of deep vein thrombosis was 42.1%, while pulmonary embolism occurred in 39.5%. However, 18.4% of the ICU patients had an occurrence of both DVT and PE. Furthermore, immobilization was identified as the most common risk factor for thromboembolic events, followed by major surgeries. More research is necessary to determine the incidence and prevalence of thromboembolic disease and its manifestations.

## Introduction

Thromboembolic events are a major complication among hospitalized patients and are associated with a high rate of morbidity and mortality [1]. Thromboembolic events are divided into two groups: venous thromboembolism (VTE) and arterial thromboembolism. The most common venous thromboembolisms are deep vein thrombosis (DVT) and pulmonary embolism (PE), while the most common arterial thromboembolisms are ischemic stroke and ischemic heart disease (IHD) [2]. A venous thromboembolism (VTE) occurs when a blood clot forms in the deep veins, particularly those of the lower extremities. Blood flow is obstructed, causing symptoms such as edema, pain, and discoloration. An embolism occurs when one of these clots becomes loosened, migrates, and occludes another blood vessel. Embolisms, and PEs, in particular, are the most prevalent sequelae of venous thrombosis [3]. VTE is considered a chronic disease, affecting about 10 million people worldwide each year [4], although VTEs significantly increase morbidity and mortality among hospitalized patients [5]. Critically ill patients are at higher risk of developing a VTE, as they have both premorbid conditions and risk factors associated with procedures commonly undertaken in intensive care, such as mechanical ventilation, central venous catheterization, sedation, invasive testing and procedures, and immobilization [3,5,6]. This study aims to assess the prevalence of thromboembolic events among intensive care unit (ICU) patients in the Al-Qassim region of Saudi Arabia.

DVTs and PEs affect between 300,000 and 600,000 Americans each year [7]. The incidence of VTE in patients who do not receive any type of prophylaxis ranges between 10% and 80%. Even with prophylaxis, patients admitted to ICUs are classified as high-risk [3]. VTE, and particularly PE, is underdiagnosed in the ICU because it is clinically silent, particularly in mechanically ventilated and sedated patients. Thromboembolic events that develop in the ICU may be difficult to detect since they can be similar to other illnesses [6]. Evidence suggests that patients with suspected DVT or PE should undergo a diagnostic assessment that includes a clinical pretest probability assessment, imaging, and a D-dimer test. VTE must be properly diagnosed and promptly managed with anticoagulant medications to avoid recurrence and possible complications [8].

An old cross-sectional retrospective study conducted in Jeddah, Saudi Arabia, examined all cases of DVT and PE admitted to King Abdulaziz University Hospital from January 1994 to March 1999 to assess the incidence, risk factors, diagnostic modalities, and treatment of DVTs and PEs. A total of 75 patients with a mean age of 44.16 years (standard deviation (SD): 14.5 years) and a male/female ratio of 1:2 were diagnosed with DVT. Doppler ultrasound (US) was used for diagnosis in 56 (75%) of the 75 patients. PE as a complication of DVT developed in 24 (32%) patients. The most common risk factor (prolonged immobilization) was present in 17 (23%) patients. All patients were treated with a conventional course of heparin, followed by warfarin. They concluded that subcutaneous heparin should be administered as preventive anticoagulation to postoperative patients who are expected to be immobilized for a prolonged period of time. Also, people with recurrent deep vein thrombosis or those with a positive family history should undergo thrombophilia screening [9].

We maintain that it would be beneficial to further test the prevalence, diagnostic modalities, management plan, causes, and risk factors of thromboembolic events. This study aims to assess the prevalence of thromboembolic events among ICU patients in the Al-Qassim region of Saudi Arabia.

## Materials and methods

This is a cross-sectional retrospective observational study of patients admitted to King Fahad Specialist Hospital in Buraydah, Saudi Arabia, between July 2020 and June 2022. To assess the prevalence, diagnostic modalities, management plan, causes, and risk factors of thromboembolic events, all patients admitted to the ICU with a thrombotic event were included. Patients with an ICU stay of less than two days were excluded.

Inclusion criteria

The inclusion criteria were as follows: ICU patients, male or female, patients hospitalized for two or more days in the ICU, and Saudis and non-Saudis.

Exclusion criteria

The exclusion criteria were as follows: non-ICU patients and patients hospitalized for less than two days in the ICU.

We collected data on age, gender, nationality, height, weight, and body mass index (BMI). We also measured the length of ICU stay, type of thrombotic event, diagnostic modalities used, risk factors, type of prophylaxis used, and treatment. Factors such as mechanical ventilation status, Glasgow Coma Scale (GCS) score, diagnosis, associated comorbidities, and mortality were also recorded. Data were collected from the clinical notes and patient files of King Fahad Specialist Hospital.

All data were analyzed using Statistical Package for the Social Sciences (SPSS) version 26 (IBM SPSS Statistics, Armonk, NY, USA). We applied descriptive statistical methods using continuous variables such as mean, standard deviation (SD), range, and median, as well as categorical variables of proportion and frequency. Furthermore, we tested statistical significance among various cross-tabulated groups by employing well-known statistical tests, including Fisher’s exact test and the chi-squared (χ2) test. We utilized the independent parametric t-test and the nonparametric Mann-Whitney U test for continuous variables to determine associated factors. We set the value of statistical significance to p<0.05. Ethical consent was acquired from the Qassim Region Research Ethics Committee (QREC) with the following approval code: 1443-830910. We guaranteed the anonymity and the protection of privacy of all study participants.

## Results

This study analyzed 38 patients (Table 1). The mean age of the patients was 60.7 (SD: 23.9) years with more than half (52.6%) being males. Saudi nationality constitutes most of the patients (89.5%). The mean value of BMI was 26 (SD: 4.55) kg/m^2^, while the median value of ICU stay was five days. The most common type of thrombotic event was DVT (42.1%), and the most common diagnosis was respiratory distress (28.9%).

**Table 1:** Baseline characteristics of the patients (n=38) BMI: body mass index; ICU: intensive care unit; Min: minimum; Max: maximum; N: number of sample; SD: standard deviation; DVT: deep vein thrombosis; PE: pulmonary embolism; COVID-19: coronavirus disease 2019

Study variables	N (%)
Age in years (mean±SD)	60.7±23.9
Gender	
Male	20 (52.6%)
Female	18 (47.4%)
Nationality	
Saudi	34 (89.5%)
Non-Saudi	4 (10.5%)
BMI (mean±SD)	26.0±4.55
ICU stay in days (median (min-max))	5.00 (2-40)
Type of thrombotic events	
DVT	16 (42.1%)
PE	15 (39.5%)
Both	7 (18.4%)
Diagnosis	
Multiple trauma	1 (2.7%)
Respiratory distress	11 (28.9%)
COVID-19	4 (10.5%)
Stroke	3 (7.9%)
Sepsis	7 (18.4%)
Gastrointestinal bleeding	1 (2.7%)
Other diagnoses	11 (28.9%)

**Figure 1:**
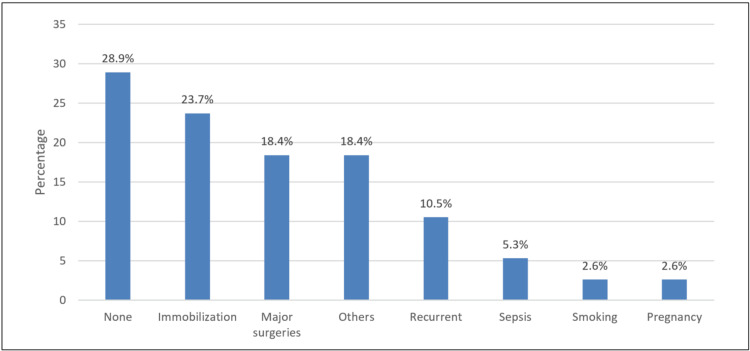
Risk factor for thrombolytic event

In Figure 1, the most common risk factor for the thrombolytic event was immobilization (23.7%), followed by major surgeries (18.4%) and recurrent thromboembolic events (10.5%).

**Figure 2:**
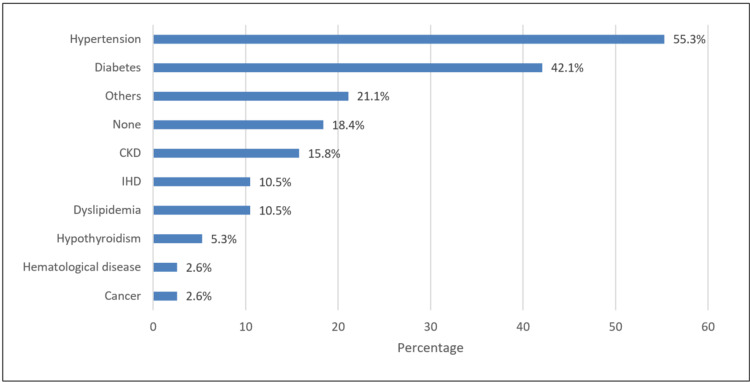
Associated chronic disease CKD: chronic kidney disease; IHD: ischemic heart disease

In Figure 2, the most commonly known associated chronic disease was hypertension (55.3%), followed by diabetes (42.1%) and chronic kidney disease (CKD) (15.8%).

Regarding the treatment and outcome of the patients (Table 2), the prevalence of patients who received mechanical ventilation was 39.5%. Of those who received mechanical ventilation, the median days of mechanical ventilation use were four days. Doppler ultrasound (US) was the most dominant imaging method (55.3%), the most commonly prescribed prophylaxis was aspirin (21%), and the most commonly used treatment was apixaban (44.7%). Mortality rates were recorded in 39.5% of patients.

**Table 2:** Treatment and outcome of the patients (n=38) ^†^Some patients underwent multiple treatments. N: number of patients; Min: minimum; Max: maximum; CT: computed tomography; CT w/c:* *computed tomography with contrast; US: ultrasound; LMWH: low-molecular-weight heparin

Variables	N (%)
Mechanical ventilation	
Yes	15 (39.5%)
No	23 (60.5%)
Duration of mechanical ventilation in days (median (min-max))	4.0 (2.0-16.0)
Imaging	
CT angiogram pulmonary	14 (34.1%)
CT of the chest w/c	2 (5.3%)
CT of the chest, lung perfusion study	2 (5.3%)
Doppler US	21 (55.3%)
Type of prophylaxis	
LMWH	2 (5.3%)
Heparin	3 (7.9%)
Aspirin	8 (21%)
No specific prophylaxis	25 (65.8%)
Treatment^†^	
Warfarin	1 (2.6%)
LMWH	11 (28.9%)
Heparin	6 (15.8%)
Surgical intervention	3 (7.9%)
Radiological intervention	3 (7.9%)
Apixaban	17 (44.7%)
Others	3 (7.9%)
Death	
Yes	15 (39.5%)
No	23 (60.5%)

When measuring the relationship between the type of thrombotic events and the baseline characteristics, treatment, and outcome (Table 3), it was revealed that the use of heparin as treatment was more associated with DVT cases (p=0.043). The rest of the variables such as age in year, gender, BMI, ICU stay, risk factors, associated chronic disease, mechanical ventilation, death, and GCS score did not show a significant relationship in the incidences of DVT and PE (all: p>0.05).

**Table 3:** Relationship between the type of thrombotic events and the baseline characteristics, its treatment, and outcome (n=31)* *Seven patients who have both DVT and PE were excluded from the analysis. ^§^P-value has been calculated using Fisher’s exact test. ^¥^P-value has been calculated using an independent sample t-test. ‡P-value has been calculated using the Mann-Whitney test. **Significant at p<0.05. N: number of patients; BMI: body mass index; Min: minimum; Max: maximum; ICU: intensive care unit; CKD: chronic kidney disease; IHD: ischemic heart disease; LMWH: low-molecular-weight heparin; GCS: Glasgow Coma Scale

Factor	Thrombotic events		P-value^§^
	DVT (N (%))^(n=16)^	PE (N (%))^(n=15)^	
Age in years (mean±SD)	58.4±26.0	65.4±19.9	0.409
Gender			
Male	9 (56.3%)	6 (40%)	0.479
Female	7 (43.8%)	9 (60%)	
BMI (mean±SD)	26.3±4.51	26.2±3.51	0.938
ICU stay in days (median (min-max))	5.5 (2-40)	7.0 (2-30)	0.858
Risk factors			
Sepsis	0	2 (13.3%)	0.226
Recurrent	2 (12.5%)	1 (6.7%)	1.000
Smoking	1 (6.3%)	0	1.000
Immobilization	3 (18.8%)	4 (26.7%)	0.685
Major surgeries	2 (12.5%)	3 (20%)	0.654
Pregnancy	0	1 (6.7%)	0.484
Others	3 (18.8%)	3 (20%)	1.000
Associated comorbidities			
Hypertension	10 (62.5%)	8 (53.3%)	0.722
Diabetes	6 (37.5%)	7 (46.7%)	0.605
CKD	3 (18.8%)	2 (13.2%)	1.000
Dyslipidemia	1 (6.3%)	2 (13.3%)	0.600
Hypothyroidism	0	2 (13.3%)	0.226
Cancer	1 (6%)	0	1.000
Hematological disease	0	1 (6.7%)	0.484
IHD	3 (18.8%)	1 (6.7%)	0.600
Others	4 (25%)	4 (26.7%)	1.000
Mechanical ventilation			
Yes	8 (50%)	5 (33.3%)	0.473
No	8 (50%)	10 (66.7%)	
Treatment			
LMWH	4 (25%)	6 (40%)	0.458
Heparin	5 (31.3%)	0	0.043**
Surgical intervention	1 (6.3%)	1 (6.7%)	1.000
Radiological intervention	0	2 (13.3%)	0.226
Apixaban	7 (43.8%)	7 (46.7%)	1.000
Death			
Yes	7 (43.8%)	4 (26.7%)	0.458
No	9 (56.3%)	11 (73.3%)	
GCS			
Mild	9 (60%)	8 (61.5%)	1.000
Moderate	3 (20%)	2 (15.4%)	
Severe	3 (20%)	3 (23.1%)	

## Discussion

This study investigates the prevalence of thromboembolic events among ICU patients in the region of Al-Qassim, Saudi Arabia. Of the 38 patients included in the study, DVT occurred in 42.1%, PE occurred in 39.5%, and DVT and PE occurred together in 18.4%. These results are higher than those of the study conducted in Jeddah [9], which showed that the incidence of DVT was 13.5% in hypertensive patients. A lower incidence of DVT and PE was also documented in Norway [10], where findings indicated that, among 27% of patients with VTE, 21% had DVT and 6% had PE. In Canada [11], the incidence of DVT was relatively low, at only 2.7%, upon ICU admission (although this increased to 9.6% during ICU stay), while in the Netherlands [12], the cumulative incidence of VTE was 31%, with 27% of the cases confirmed by CT pulmonary angiogram (CTPA) or ultrasonography. PEs accounted for 81% of thrombotic complications, higher than the incidence rate of PE in the general population.

After excluding patients with the combined disease (DVT and PE), the data in this study suggest that only the use of heparin medication was found to have a direct relationship with thrombotic events. The prevalence of patients using heparin medication was significantly higher in DVT patients (p=0.043). Other studied variables, including age (in years), gender, BMI, length of ICU stay, associated risk factors, associated comorbidities, mechanical ventilation status, mortality rates, and GCS score, had similar incidences of DVT and PE. In China [13], among elderly patients, several significant factors of VTE were reported, including being of the male sex, immobilization, sepsis, history of DVT, diabetes mellitus (DM), pneumonia, congenital heart defects (CHDs), heart failure, low PaO2, mechanical ventilation status, concurrent chemoradiotherapy (CCRT), and glucocorticoid treatment. A Canadian study [11] found that a personal or family history of VTE, end-stage renal failure, platelet transfusion, and vasopressor usage were independent significant predictors of VTE development in the ICU and that DVT incidence was associated with a longer duration of stay in the ICU, mechanical ventilation status, and hospitalization. However, a study conducted in Jeddah [9] found no significant differences in the development of DVT in relation to age, gender, comorbidities, and other studied factors, which did not coincide with previous reports.

Moreover, a study by Minet et al. [6] found that severely ill patients are at increased risk of VTE due to risk factors associated with ICU stay, such as immobilization, sedation, central venous catheterization, and vasopressor usage. In India [14], the most common risk factors recorded were advanced age (>60 years) (57.1%), extended immobility (50.6%), respiratory disorders (41.6%), and acute infectious disease (36.2%). In our study, the most commonly cited risk factor for thromboembolic events was immobilization (23.7%), followed by major surgeries (18.4%) and recurrent thromboembolic events (10.5%). Other observed risk factors were sepsis (5.3%), smoking (2.6%), and pregnancy (2.6%). More investigations are needed for further insight into the general risk factors of patients with thromboembolic events.

Aspirin was the most commonly used prophylactic for thrombotic events. In India [14], two of the most prominent mechanical and pharmacological prophylaxes administered to nearly all (98.3%) high-risk VTE patients were early mobilization (44.3%) and LMWH (66.2%), while 80.6% were given thromboprophylaxis, according to the American College of Clinical Pharmacy (ACCP) guidelines. An investigation by Cook et al. [5], which involved 29 ICU directors in Canadian university-affiliated hospitals, reported that unfractionated heparin was the predominant VTE prophylactic administered among ICU patients. The study also noted that the diagnosis of PE usually relied on spiral chest CT scans and ventilation, while for DVT, ultrasound was the most common imaging method used. The directors’ final suggestion was that further studies be conducted among critically ill subjects to establish the risks and benefits of investigating VTE and the cost-effectiveness of prophylaxis among ICU patients.

Regarding the treatment of patients with thromboembolic events, apixaban was the therapeutic of choice for treatment. Doppler ultrasound was assumed to be the most trusted imaging method (55.3%).

## Conclusions

Immobilization was identified as the most common risk factor for thromboembolic events. Major surgery was identified as the second most common. Doppler ultrasound was the most effective imaging method for the detection of the disease, while aspirin was the most commonly administered thromboprophylaxis. More research is warranted to determine the incidence rate of thromboembolic disease and its manifestations.
